# Efficacy of Postprandial Exercise in Mitigating Glycemic Responses in Overweight Individuals and Individuals with Obesity and Type 2 Diabetes—A Systematic Review and Meta-Analysis

**DOI:** 10.3390/nu15204489

**Published:** 2023-10-23

**Authors:** Jie Kang, Brian M. Fardman, Nicholas A. Ratamess, Avery D. Faigenbaum, Jill A. Bush

**Affiliations:** 1 Department of Kinesiology and Health Sciences, The College of New Jersey, Ewing, NJ 08618, USA; ratamess@tcnj.edu (N.A.R.); faigenba@tcnj.edu (A.D.F.); wallacej@tcnj.edu (J.A.B.); 2 Rowan-Virtua School of Osteopathic Medicine, Stratford, NJ 08084, USA; fardma54@rowan.edu

**Keywords:** glycemic control, hyperglycemia, exercise protocols, exercise timing, metabolic disorders

## Abstract

Studies investigating the acute effect of postprandial exercise (PPE) on glucose responses exhibit significant heterogeneity in terms of participant demographic, exercise protocol, and exercise timing post-meal. As such, this study aimed to further analyze the existing literature on the impact of PPE on glycemic control in overweight individuals and individuals with obesity and type 2 diabetes (T2DM). A literature search was conducted through databases such as PubMed, CINAHL, and Google Scholar. Thirty-one original research studies that met the inclusion criteria were selected. A random-effect meta-analysis was performed to compare postprandial glucose area under the curve (AUC) and 24 h mean glucose levels between PPE and the time-matched no-exercise control (CON). Subgroup analyses were conducted to explore whether the glucose-lowering effect of PPE could be influenced by exercise duration, exercise timing post-meal, and the disease status of participants. This study revealed a significantly reduced glucose AUC (Hedges’ g = −0.317; SE = 0.057; *p* < 0.05) and 24 h mean glucose levels (Hedges’ g = −0.328; SE = 0.062; *p* < 0.05) following PPE compared to CON. The reduction in glucose AUC was greater (*p* < 0.05) following PPE lasting >30 min compared to ≤30 min. The reduction in 24 h mean glucose levels was also greater (*p* < 0.05) following PPE for ≥60 min compared to <60 min post-meal and in those with T2DM compared to those without T2DM. PPE offers a viable approach for glucose management and can be performed in various forms so long as exercise duration is sufficient. The glucose-lowering effect of PPE may be further enhanced by initiating it after the first hour post-meal. PPE is a promising strategy, particularly for patients with T2DM. This manuscript is registered with Research Registry (UIN: reviewregistry1693).

## 1. Introduction

Persistent postprandial hyperglycemia is an important risk factor for type 2 diabetes mellitus (T2DM) and its complications, which include cardiovascular diseases and mortality [1,2,3]. In fact, postprandial glucose is often considered a more dynamic indicator of metabolic health and cardiovascular disease risk compared to glycated hemoglobin (HbA1c) or fasting glucose [4,5,6]. Impaired postprandial glycemia is also associated with inflammation, oxidative stress, and impaired endothelial function [7]. Longitudinal studies have demonstrated that reducing postprandial glucose not only improves glycemic control but also reduces cardiovascular disease risk in patients with T2DM [8,9]. Given the recurrent exposure to postprandial hyperglycemia throughout the day, the postprandial phase has emerged as a pivotal focus for the prevention and treatment of diabetic conditions.

Exercise is vital in the prevention and treatment of T2DM. Both endurance and resistance exercise can acutely enhance insulin sensitivity and glucose tolerance [10,11,12]. These improvements have been attributed to the activation of the skeletal muscle glucose transporter system [13], the depletion of muscle and liver glycogen stores [14,15], and/or increased skeletal muscle blood flow [16] following the cessation of exercise. In adults with prediabetes or T2DM, a net reduction in blood glucose concentration during exercise is usually observed [17]. It has been suggested that strategies to combat T2DM should consider exercise after meals to more effectively tame postprandial glucose excursions [18,19]. When exercise is performed postprandially, both contraction- and insulin-mediated glucose uptake are stimulated, resulting in an additive effect on skeletal muscle glucose uptake [20]. Exercise after a meal is also linked to an elevated insulin-to-glucagon ratio, which can suppress hepatic glucose output, thereby lowering blood glucose levels [21,22].

A key objective of adopting postprandial exercise (PPE) is to effectively balance the rate of meal-derived glucose entering the bloodstream with the rate at which exercise utilizes this fuel. In this context, the impact that exercise has on postprandial glucose profile will depend in part on the time elapsed between the start of the preceding meal and the initiation of subsequent exercise. If exercise occurs too soon while meal-derived glucose levels are still low, or if it occurs too late, i.e., beyond the peak blood glucose period, the opportunity to effectively blunt postprandial hyperglycemia can be lost [23]. Recent reviews suggest that exercise initiated 30 min after a meal may produce the greatest improvements in glycemic control for individuals with T2DM [24,25]. However, this view is based on a limited number of studies that mainly compare the effect of pre- and post-meal exercise on glycemic responses in individuals with T2DM [26,27,28]. Studies involving an exercise regimen 60 min or more after a meal have also shown a significant reduction in glucose response in this population [29,30,31,32,33,34,35,36].

While managing postprandial hyperglycemia holds crucial significance in treating and preventing T2DM, exercise strategies during this phase that could be most effective in mitigating blood glucose levels have yet to be fully established. Among the studies exploring the acute effect of postprandial exercise on glycemic control, substantial heterogeneity exists in aspects such as participant demographics, exercise protocols, and exercise timing post-meal, making it challenging in directly applying the findings. Among the studies in the literature, there are also disparities in how postprandial glycemic responses have been determined. Glycemic responses following PPE can be assessed by quantifying the area under the curve (AUC) for glucose a few hours after exercise or by determining the mean glucose concentration over a 24 h period that includes the exercise treatment. These two measures, however, do not always yield the same results [29,37,38,39].

Therefore, the aim of this study was to comprehensively examine the available literature on the impact of PPE on glycemic control, including assessments of both the glucose AUC and the 24 h mean glucose concentration. Subgroup analyses were included to further explore whether the glucose-lowering effect of PPE could be influenced by exercise duration, exercise timing post-meal, and participants’ disease status. By pooling and stratifying data from multiple studies via a meta-analytical approach, this quantitative review could provide more compelling evidence on how PPE may be best arranged tactically to minimize meal-induced glucose surges in overweight adults and adults with obesity and T2DM.

## 2. Methods

### 2.1. Search Strategy

This review followed the Preferred Reporting Items for Systematic Reviews and Meta-Analyses (PRISMA) guidelines [40]. A literature search was conducted through electronic databases such as PubMed, CINAHL, and Google Scholar; our search began in July 2022 and ended in June 2023. Only English language articles were retrieved. The search was restricted to peer-reviewed publications reporting the acute effects of postprandial exercise on glycemic responses in human subject experimentation since 1970. The search was performed by using various combinations of the key words or phrases concerning (1) the exercise treatment (such as “postprandial exercise”, “post-meal exercise”, and “exercise after eating”) and (2) the outcome measures (such as “glycemia”, “hyperglycemia”, and “blood glucose concentrations”). Articles were also identified by a manual search via cross-referencing the original research papers, review articles, and lay press publications. The search results were imported into the EndNote software program (EndNote v20) to organize references and remove duplicates.

### 2.2. Inclusion and Exclusion Criteria

The studies that were chosen for further analysis met the following inclusion criteria: (a) participants were 18 years of age or older and overweight (i.e., BMI ≥ 25 kg·m^−2^), obese (i.e., BMI ≥ 30 kg·m^−2^) or diagnosed with T2DM (i.e., HbA1c ≥ 6.5%); (b) studies involved exercise protocols that were initiated after a meal and measured the acute effect of postprandial exercise on glycemic responses; (c) studies included a control condition of no exercise (CON) conducted in the same postprandial period on a different day; and (d) glycemic responses were assessed via AUC for glucose post exercise and/or by the mean glucose concentration over a 24 h period that included the experimental treatment. In accordance with these inclusion criteria, we excluded studies that (a) used animal models, healthy, normal-weight participants, or special populations such as adolescents, pregnant women, and patients with type 1 diabetes; (b) employed a longitudinal study design involving physical training; (c) failed to use a separate and time-matched control condition; (d) implemented an exercise protocol outside of postprandial period; and (e) assessed other postprandial parameters, such as blood lipid levels, substrate oxidation, hormonal responses, antioxidant status, and immune function. Each article was independently screened and approved by two authors based on these inclusion and exclusion criteria.

### 2.3. Assessment of Methodological Quality

To assess the methodological quality of the included studies, a 7-question checklist was created according to the National Heart, Lung, and Blood Institute (NHLBI) Quality Assessment Tool for Observational Cohort and Cross-Sectional Studies (https://www.nhlbi.nih.gov/health-topics/study-quality-assessment-tools, accessed on 15 July 2022). The 7 questions in this checklist are as follows: (1) Was the research question or objective in this paper clearly stated? (2) Was the study population clearly defined and characterized? (3) Were inclusion and exclusion criteria for being in the study prespecified and applied uniformly to all participants? (4) Was sample size justification, power calculation, or effect size estimate provided? (5) Were the exposure measures (independent variables) clearly defined and applied consistently to all participants? (6) Were the outcome measures (dependent variables) clearly defined and measured consistently across all participants? (7) Were potential confounding variables such as medications, diet, and/or physical activity prior to experimental trials properly controlled? The assessment was made independently by two coauthors.

### 2.4. Data Extraction

Extracted data included (1) study authors and year of publication, (2) participants’ characteristics, (3) PPE protocols, (4) test meal profiles, (5) outcome variables, and (6) major findings. The outcome variables included the postprandial glucose excursions—measured by glucose AUC or incremental AUC (if AUC is not available) and 24 h mean glucose concentrations. The glucose AUC measures the more immediate glucose response, while the 24 h mean glucose concentration provides a more complete picture of glycemic control throughout the day [41,42]. If the studies presented standard errors (SEs), they were converted to standard deviations (SDs) using the formula of SE times the square root of sample size [43]. If the studies only presented 95% confidence intervals, SDs were calculated as the confidence interval length divided by 3.92 and multiplied by the square root of the sample size [43]. When results were only shown in graphs and the corresponding authors could not be reached, they were extracted using the Web Plot Digitizer (Web Plot Digitizer V3.11). Data extraction was independently performed by two authors and crosschecked for accuracy.

### 2.5. Statistical Analysis

Meta-analysis was conducted to compare glycemic responses between PPE and CON using the Statistical Package for Social Sciences Version 28 (SPSS v28, SPSS Inc., IBM Corp, Armonk, NY, USA). Additional comparisons of glycemic responses were made between high-intensity interval exercise (HIIE) and continuous moderate-intensity exercise (CMIE) and between exercise post-meal and pre-meal. To facilitate subgroup analyses, the chosen studies were further dichotomized into groups based on (1) exercise duration (i.e., ≤30 min vs. >30 min), (2) exercise timing post-meal (i.e., <60 min vs. ≥60 min), and (3) participants’ disease status (i.e., with vs. without T2DM). As a result, three separate subgroup analyses were performed for each of these three factors. The random-effects model was chosen due to the expected heterogeneity among the chosen studies. The effect size of comparison was determined by Hedge’s *g* to reduce the potential bias associated with small sample sizes [44]. To justify the adequacy of combining studies, the study homogeneity was evaluated by using the *I^2^* static and interpreted as “high” if *I*^2^ was < 50%, “moderate” if *I*^2^ was between 50 and 75%, and “low” if *I*^2^ was > 75% [45]. For each of the three subgroup analyses, a chi-square-based heterogeneity test was used to detect if the effect size associated with one subgroup significantly differed from the others. In addition, publication bias was evaluated using the Egger’s test in combination with the Trim and Fill procedure, which adjusts the effect size based on potential theoretical missing studies if such occurrences were to happen [46]. For all analyses, the statistical significance threshold was set at *p* ≤ 0.05.

## 3. Results

### 3.1. Study Selection

**Figure 1:**
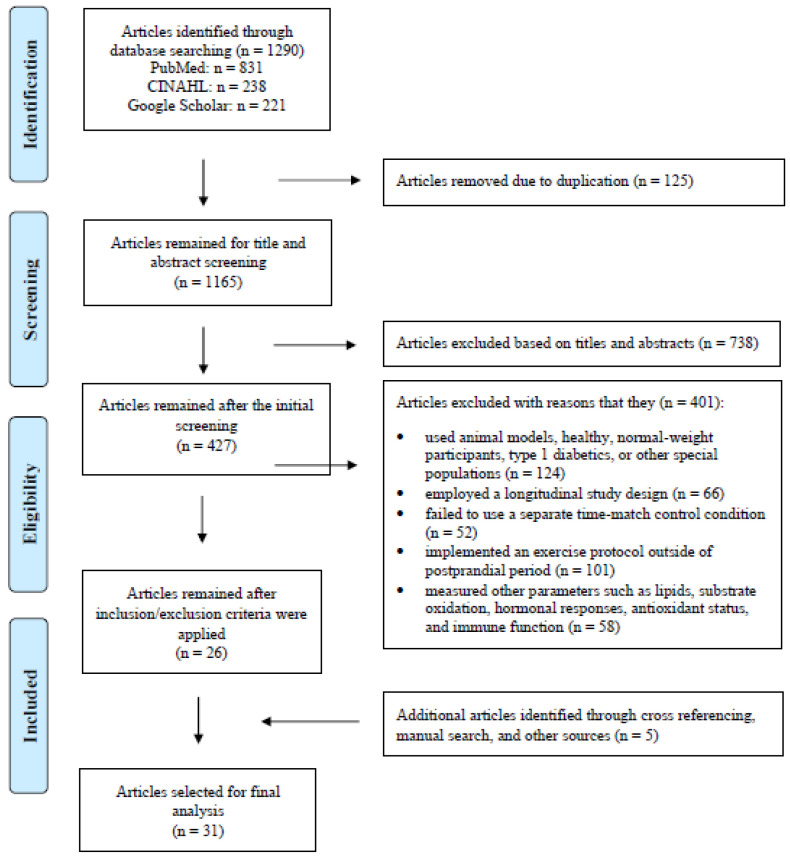
PRISMA Flow diagram illustrating the selection process for articles included in this systematic review and meta-analysis.

The initial searches of the aforementioned databases identified 1290 potential full-text, peer-reviewed original research articles. After removing the duplicates and the studies that did not fit the research objective based on their titles and abstracts, 427 articles remained and were subjected to further screening. When both the inclusion and exclusion criteria were applied in conjunction with the articles retrieved from our manual search, 31 articles were selected for the final analysis (Figure 1).

### 3.2. Study Characteristics

The experimental details of the 31 chosen studies are presented in Table 1. Collectively, these studies included a total of 516 participants aged from 21 to 69 years old, and ~77% of them were men. The average body mass index (BMI) of all participants in all studies combined was 30 kg·m^−2^. Of the 31 selected studies, 21 recruited patients with T2DM [26,28,29,30,31,32,33,34,35,36,37,38,47,48,49,50,51,52,53,54,55], while the remaining 10 studies involved overweight and obese participants [39,56,57,58,59,60,61,62,63,64]. Of those 21 studies on patients with T2DM, 3 included patients that were treated with exogenous insulin [34,35,36], while the rest allowed their participants to use glucose-lowering medications but instructed them to keep the same dosage throughout the study period. All studies employed a cross-over design in which the same participants underwent both PPE and CON on separate days in a randomized or counterbalanced order. This research setup helps control for diurnal variations and minimize carry-over effects, thus enhancing the internal validity of the study.

The exercise protocols employed in the selected studies were predominantly aerobic (Table 1). Exercise intensity varied between 3 METs and 90% VO_2_max, with durations ranging from 3 to 60 min. The exercise modalities encompassed activities such as brisk walking with and without inclines, stair climbing and descending, stationary cycling, and resistance exercise. Six studies compared glycemic responses between HIIE and CMIE [32,52,54,60,62,63]. In these studies, the average intensity and duration were approximately 90% VO_2_max and 28 min, respectively, for HIIE and 60% VO_2_max and 46 min, respectively, for CMIE. Two studies examined glycemic responses to postprandial resistance exercise [28,33], and two compared such responses between resistance and aerobic exercise [35,47]. Resistance exercises were implemented using medicine balls, elastic bands, calisthenics, and resistance machines, and the intensity and duration of these activities were between 30–70% of one-repetition maximum (1 RM) and between 15–45 min, respectively. Five studies also included a pre-meal exercise trial in which exercise was performed in the postabsorptive or fasting state in addition to the postprandial period [26,28,54,57,61].

All but one study provided specific nutrient information either on the test meal or for the entire day when the experimental trial was conducted (Table 1). On average, participants consumed 507 kcal per meal or 2217 kcal per day, with 59% of the caloric intake being derived from carbohydrates. Most studies were conducted in the morning following a standardized breakfast, with only six studies being performed in the afternoon or evening. The selected studies exhibited a notable range in the time elapsed between the test meal and the subsequent exercise, spanning from 20 to 150 min.

All the studies assessed the impact of postprandial exercise by analyzing glucose AUC or iAUC within a 1 to 6 h timeframe and/or by determining the average glucose concentration over a 24 h period in response to PPE. Of the chosen studies, 17 utilized continuous glucose monitoring to measure glucose responses, while the remaining studies employed the conventional blood sampling technique. In the majority of the studies, the experimental treatment, whether PPE or CON, took place within the glucose measurement period. However, two studies did not record blood glucose levels until immediately after exercise cessation [32,56].

### 3.3. Methodological Quality and Publication Bias

Detailed results of quality assessment for the included studies are presented in Table 2. Overall, 26 of the 31 studies received a score of 6 or higher, while the average score of all studies combined based on the seven-question checklist was 6.1, exceeding the threshold rating for high quality, i.e., 85% [65]. The most common concern with the chosen studies related to a lack of sample size justification, and this was followed by an inadequate elaboration regarding the criteria used in relation to participant recruitment. Egger’s test indicated that publication bias was insignificant for both the postprandial glucose AUC and 24 h mean glucose concentrations. Moreover, the Trim-and-Fill analysis, which was used to impute potentially missing studies, suggested that no adjustment to the effect size was deemed necessary for either of these two variables.

### 3.4. Results of Meta-Analysis

#### 3.4.1. PPE vs. CON on Postprandial Glucose AUC

**Figure 2:**
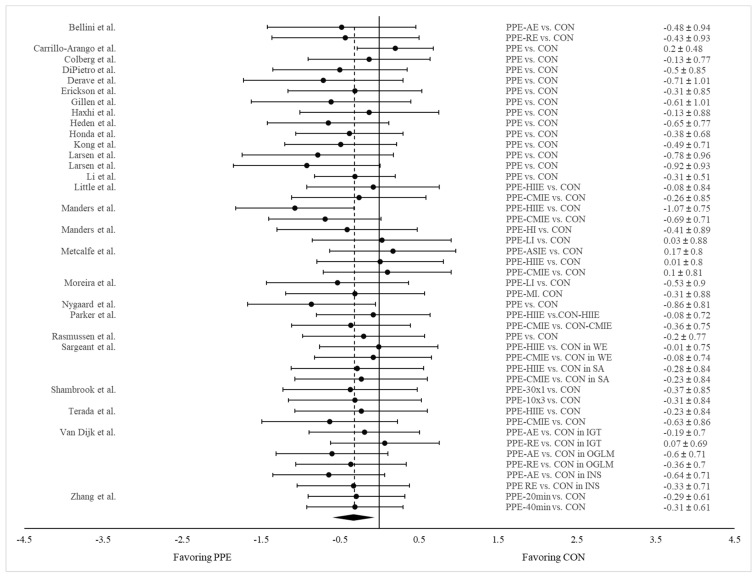
Forest plot showing comparisons of glucose AUC between PPE and CON. The *X*-axis denotes the effect size derived from the random-effect meta-analysis. The data in the center-right column specify the conditions under which PPE and CON were compared, while the far-right column provides the effect size and 95% confidence interval (CI) for each comparison. The diamond at the bottom represents the pooled effect size (Hedge’s g = −0.317; SE = 0.057; 95% CI = −0.432, −0.201; *p* < 0.001) based on all the studies combined.

Twenty-seven studies reported glycemic response as measured by glucose AUC or iAUC for both PPE and CON (Table 1). Twelve of them employed more than one exercise treatment (e.g., AE and RE, HIIE, and CMIE) and exercises with varying intensities, durations, or frequencies. Two studies examined exercise effects in participants differing in ethnic background or disease status. Consequently, a total 46 pairs of data were extracted and pooled for comparison between PPE and CON. Our meta-analysis revealed significantly lower glucose AUC in PPE compared to CON (Hedge’s g = −0.317; SE = 0.057; 95% CI = −0.432, −0.201; *p* < 0.001) (Figure 2). *I*^2^ equaled zero, suggesting a high level of consistency regarding this measure across the studies.

#### 3.4.2. PPE vs. CON on 24 h Mean Glucose Concentration

**Figure 3:**
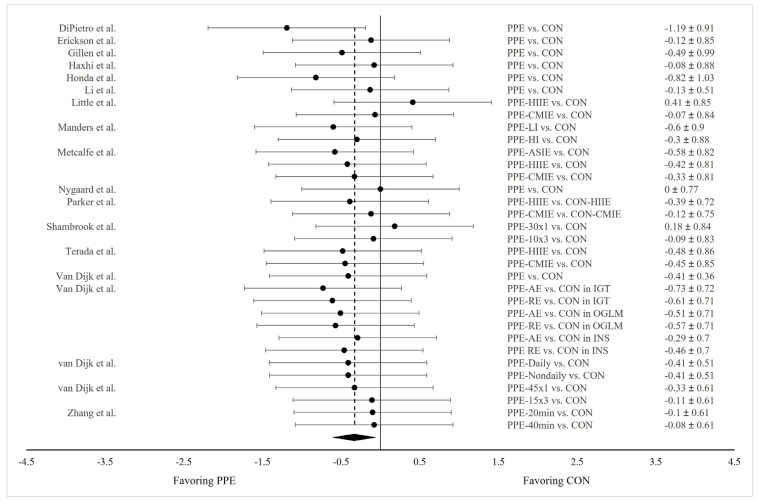
Forest plot showing comparisons of 24 h mean glucose concentrations between PPE and CON. The *X*-axis denotes effect size derived from the random-effect meta-analysis. The data in the center-right column specify the conditions under which PPE and CON were compared, while the far-right column provides the effect size and 95% confidence interval (CI) for each comparison. The diamond at the bottom represents the pooled effect size (Hedge’s g = −0.328; SE = 0.062; 95% CI = −0.453, −0.203; *p* < 0.001) based on all the studies combined.

Eighteen studies examined ~24 h mean glucose concentrations in response to PPE, and eight of them employed more than one exercise treatment (e.g., AE and RE, HIIE, and CMIE) and exercises with varying intensities, durations, or frequencies. One study examined exercise effects in participants differing in disease status. Therefore, a total 33 pairs of data were extracted and pooled for comparison between PPE and CON. Our meta-analysis revealed significantly lower 24 h mean glucose concentrations in PPE compared to CON (Hedge’s g = −0.328; SE = 0.062; 95% CI = −0.453, −0.203; *p* < 0.001) (Figure 3). *I*^2^ equaled zero, suggesting a high level of consistency regarding this measure across the studies.

#### 3.4.3. HIIE vs. CMIE on Both Post Prandial Glucose AUC and 24 h Mean Glucose Concentration

**Figure 4:**
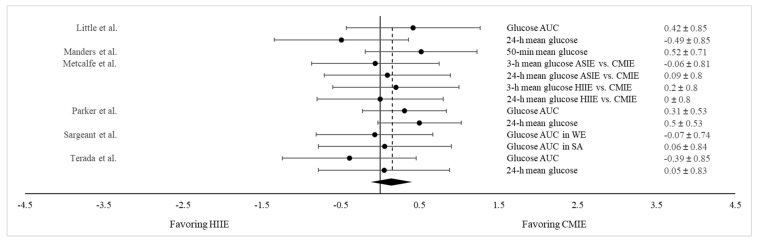
Forest plot showing comparisons of glycemic responses (as measured by both the glucose AUC and the 24 h mean glucose level between HIIE and CMIE). The *X*-axis denotes effect size derived from the random-effect meta-analysis. The data in the center-right column specify the conditions under which PPE and CON were compared, while the far-right column provides the effect size and 95% confidence interval (CI) for each comparison. The diamond at the bottom represents the pooled effect size (Hedge’s g = 0.152; SE = 0.104; 95% CI = −0.075, 0.397; *p* = 0.170) based on all the studies combined.

A separate meta-analysis was conducted to compare the glucose-lowering effect between HIIE and CMIE. This was accomplished by extracting results on the postprandial glucose AUC and the 24 h mean glucose concentration from the six studies that tested both the HIIE and CMIE protocols [32,52,54,60,62,63]. While all six studies measured the glucose AUC, five of them also assessed the 24 h mean glucose concentration following PPE. In addition, Metcalfe et al. [52] implemented two different HIIE protocols, and Sargeant et al. [63] examined exercise effects in participants from two different ethnic backgrounds separately. Consequently, a total of 13 pairs of data on both the postprandial glucose AUC and 24 h mean glucose concentrations were combined for comparison between HIIE and CMIE. As shown in Figure 4, there were no differences in glycemic responses between HIIE and CMIE (Hedge’s g = 0.152; SE = 0.104; 95% CI = −0.075, 0.397; *p* = 0.170). This finding suggests that both HIIE and CMIE are equally effective in mitigating postprandial hyperglycemia.

#### 3.4.4. Postprandial vs. Pre-meal Exercise on Both Postprandial Glucose AUC and 24 h Mean Glucose Concentration

**Figure 5:**
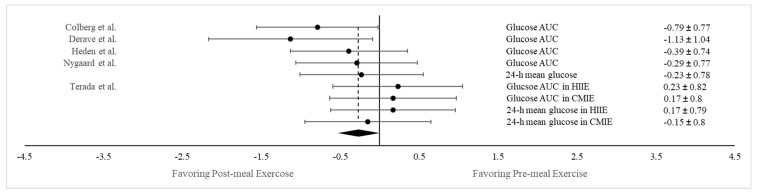
Forest plot showing comparisons of glycemic responses (as measured by both the glucose AUC and the 24 h mean glucose level between pre-meal and post-meal exercise conditions). The *X*-axis denotes effect size derived from the random-effect meta-analysis. The data in the center-right column specify the conditions under which PPE and CON were compared, while the far-right column provides the effect size and 95% confidence interval (CI) for each comparison. The diamond at the bottom represents the pooled effect size (Hedges’ g = −0.271; SE = 0.072; 95% CI = −0.357, −0.085; *p* < 0.05) based on all the studies combined.

Overall, 5 of the 31 chosen studies also incorporated a pre-meal exercise trial in addition to PPE [26,28,54,57,61]. This allowed us to also compare the blood-lowering effect of exercise between the pre- and post-meal conditions in an aggregated manner. Of these five studies, two measured both the glucose AUC and the 24 h mean glucose concentration [54,61], and one of these two also employed two exercise conditions: HIIE and CMIE [54]. Hence, a total of nine pairs of data on both the glucose AUC and the 24 h mean glucose concentration were combined for comparison between the pre- and post-meal conditions. As shown in Figure 5, a significantly greater reduction in glycemic response was noted following PPE compared to the pre-meal condition (Hedges’ g = −0.271; SE = 0.072; 95% CI = −0.357, −0.085; *p* < 0.05), suggesting that engaging in PPE is more efficacious in attenuating postprandial hyperglycemia compared to exercising before a meal.

### 3.5. Subgroup Analyses

#### 3.5.1. Results on Postprandial Glucose AUC

The 27 studies were further categorized according to (1) exercise duration (i.e., ≤30 min or >30 min), (2) exercise timing (i.e., <60 min or ≥60 min after meal), and (3) disease status (i.e., with or without T2DM). Such stratifications allow for a more precise understanding of how various factors may interact to influence metabolic outcomes of PPE. As shown in Table 3, the glucose-lowering effect was significantly less in the ≤30 min than the >30 min group (chi-square Q = 4.361, *p* < 0.05). However, the glucose-lowering effects were similar between PPE initiated <60 min and ≥60 min after a meal (chi-square Q = 0.044, *p* = 0.833) and between those with and without T2DM (chi-square Q = 1.194, *p* = 0.275) (Table 3).

#### 3.5.2. Results on 24 h Mean Glucose Concentration

Another subgroup analysis was conducted on the 24 h mean glucose levels by classifying the 18 studies based on the same criteria mentioned above. Only a modest trend towards a greater reduction in 24 h mean glucose levels in the >30 min group than the ≤30 min group was observed (chi-square Q = 1.994, *p* = 0.158) (Table 3). However, notable differences emerged in terms of exercise timing and disease status. The glucose-lowering effect was significantly smaller following PPE initiated <60 min compared to ≥60 min after a meal (chi-square Q = 4.463, *p* < 0.05) (Table 3). Moreover, participants with T2D experienced a significantly greater reduction in glucose levels than those without T2D (chi-square Q = 4.104, *p* < 0.05) (Table 3).

## 4. Discussion

This review sought to quantitatively analyze the current literature surrounding the role PPE plays in mitigating meal-induced hyperglycemia in individuals who are overweight, obese, and have T2DM. Through combining multiple studies that have investigated the acute metabolic effects of PPE, a modest yet significant reduction in blood glucose response following PPE was detected in those with and without T2DM. The reduction was evident in both the postprandial glucose AUC, which quantifies the immediate glycemic response to PPE, and the 24 h mean glucose concentration, which provides a more comprehensive assessment of glycemic control. Taken together, these findings suggest that exercise post-meal can not only lead to a transitory reduction in postprandial glucose levels but also contribute to positive glycemic control throughout the day. The underlying mechanism for the glucose-lowering effect of PPE can be attributed to the muscular activity that occurs concurrently with the buildup of glucose in the blood from the meal. When this occurs, it stimulates both contraction- and insulin-mediated glucose uptake, thereby removing glucose from the bloodstream more effectively [20]. During this time period, the insulin-to-glucagon ratio is also elevated, which could further reduce blood glucose concentration by inhibiting hepatic glucose output [23].

Exercise protocols in studies demonstrating a significant reduction in postprandial glucose AUC or 24 h mean glucose concentrations following PPE exhibited wide variations in intensity, duration, and modality. These protocols can be broadly categorized as either more vigorous shorter-duration exercises or less intense exercises for a longer duration. While the majority of studies utilized treadmill walking and stationary cycling, the glucose-lowering effects of alternative exercises, including bodyweight exercises, exercises involving medicine balls or elastic bands, and daily activities such as stair climbing, gardening, and household tasks, have also been demonstrated. In general, higher-intensity exercises are more effective in enhancing cellular glucose uptake due to their high demand for glycogen [66,67,68]. However, this evidence may not directly apply in the context of PPE, where the overall exercise volume is relatively small. Characterizing a generalized dose–response relationship for PPE is challenging due to the diverse exercise protocols used in the selected studies. Regarding exercise duration, our subgroup analysis that involved dividing studies based on PPE of ≤30 min vs. >30 min revealed a significantly larger reduction in postprandial glucose AUC associated with exercise lasting more than 30 min. It appears that although PPE may take various intensities or forms, its glucose-lowering effect can be ensured or potentially maximized by maintaining a sufficient exercise duration (i.e., 30 min or longer).

There has been a growing interest in using HIIE following meals as a time-efficient approach to counter postprandial hyperglycemia, as HIIE has been shown to enhance glucose tolerance and insulin sensitivity [69,70,71]. Given the high-intensity nature of HIIE, incorporating it into a PPE should theoretically enhance glucose disposal into skeletal muscle, reducing meal-induced glucose responses. As part of our secondary analysis, we also compared the glucose-lowering effect between HIIE and CMIE from the six studies that attempted to examine the role a more time-efficient exercise protocol plays in mitigating postprandial hyperglycemia [32,52,54,60,62,63]. On average, the intensity and duration attained were ~90% VO_2_max and ~28 min, respectively, in HIIE and ~60% VO_2_max and ~45 min, respectively, in CMIE. Notwithstanding these disparities between HIIE and CMIE, a similarity was observed in their glucose-lowering effects (as measured by the postprandial glucose AUC and the 24 h mean glucose concentrations combined). This finding is consistent with an earlier review by Borror et al. [17] and suggests that the combination or interaction of intensity and duration or the total exercise volume are the most critical factors in determining the effectiveness of PPE. Caution should be used when interpreting the results on this issue, as only six studies were used for the comparison. One should also be cognizant that despite HIIE protocols being time-efficient, their intense nature could potentially increase hepatic glucose output and worsen postprandial hyperglycemia [72,73].

Another major aim of this review was to address the temporal optimization of exercise in relation to meal consumption. Knowing an ideal time frame when exercise should commence after a meal can help further increase the metabolic benefits from the same exercise. Our secondary analysis based on the five studies that implemented an exercise trial before and after a meal revealed a significantly larger glucose-lowering effect associated with exercise being carried out post-meal [26,28,54,57,61]. This finding agrees with many earlier reports suggesting that exercise undertaken postprandially confers better glycemic control than exercise initiated before a meal [18,24]. Pre-meal exercise may not be as effective in part because exercise in the postabsorptive state has been associated with more activated counterregulatory hormones such as glucagon and epinephrine, which trigger a greater hepatic glucose output [22].

When exploring the optimal timing for initiating PPE, the answer appears to diverge widely across the selected studies, ranging from 20 min to 150 min after a meal. Earlier studies involving healthy humans suggest that commencing exercise within 30–45 min after a meal is more effective in blunting postprandial hyperglycemia because it coincides more closely with the time when the blood glucose level reaches its peak [74,75,76]. However, based on our subgroup analysis of exercise timing post-meal, the reduction in glucose AUC appears unaffected by exercise timing, that is, whether postprandial exercise is initiated within the first hour after a meal or later, it remains equally effective in eliciting a transient reduction in blood glucose levels during the postprandial period. As for the 24 h mean glucose concentration, it demonstrated a significantly greater reduction following PPE initiated ≥60 min compared to <60 min after a meal. This review targeted individuals who were overweight, obese, and diagnosed with T2DM, and nearly 70% of the chosen studies involved patients with T2DM. It has been suggested that individuals with impaired insulin action may experience a more prolonged and higher peak in postprandial glucose levels between 60 and 90 min after a meal [77,78,79]. In this context, it appears that for individuals with T2DM or insulin resistance, engaging in PPE during the second hour after a meal may allow them to maximize the glucose-lowering benefit of exercise, particularly when seeking better glycemic control throughout the day.

Our subgroup analysis that involved categorizing participants by disease status revealed that patients with T2DM exhibited a significantly greater reduction in 24 h mean glucose concentrations in response to PPE when compared to those who are overweight and obese. This finding is consistent with an early clinical trial in which individuals with higher HbA1c levels (≥7%) experienced greater absolute reductions in blood glucose concentrations following PPE [34], and it underscores the critical role of incorporating PPE into daily glycemic control strategies, particularly for individuals with more severe metabolic disorders. Blood glucose levels tend to rise more after a meal in patients with diabetes compared to non-diabetic individuals [80]. This may explain why the greater glucose-lowering effect was seen in patients with T2DM, as higher plasma glucose concentrations have been correlated with greater cellular glucose uptake following exercise even when insulin levels remain unchanged [81]. As the majority of patients considered in this review received glucose-lowering medications or exogenous insulin during the study, it is plausible that these medical interventions may have additively interacted with the exercise regimen, contributing to the more pronounced reduction in blood glucose responses observed in diabetic participants.

## 5. Strengths and Limitations

This review was based on the most recent publications concerning the benefits of postprandial exercise and included studies that employed a randomized or counterbalanced crossover design. Aggregating data from studies sharing similar designs enabled a more precise assessment of the impact of postprandial exercise on blood glucose responses. Our analysis encompassed data on both post-meal glucose AUC and the 24 h mean glucose concentration, enhancing our comprehensive understanding of how PPE could influence glycemic control over an extended period of time. The inclusion of subgroup analysis considering exercise duration, exercise timing, and disease status also enabled us to more specifically explore how this exercise approach could be optimally implemented. Furthermore, the findings from this review have clinical applicability, as the study included participants who were at risk of developing T2DM or already diagnosed with the condition.

It should be noted that this study only evaluated the acute responses of exercise, so how postprandial exercise may affect glycemic control over time warrants further investigation. Given the highly diverse exercise protocols used in the selected studies, we cannot recommend a specific optimal intensity range. As a majority of the chosen studies utilized a once-daily exercise regimen, it remains uncertain whether the glucose-lowering impact of PPE would be heightened through multiple exercise sessions distributed across the day compared to a single exercise session of equal volume. The test meals used in the studies supplied sufficient calories (~500 kcal), yet they were higher in carbohydrates, which constituted an average of ~60% of the total caloric intake. Therefore, it is important to investigate how blood glucose may respond when PPE follows meals with diverse nutrient compositions, such as those high in fat or protein. Additionally, this review did not evaluate hyperglycemia prevalence, which may not always correlate with 24 h mean glucose concentrations [82,83].

## 6. Conclusions

This analytical study examined the acute impact of postprandial exercise on glycemic responses by incorporating evaluations of both the immediate post-meal glucose AUC and the 24 h mean glucose concentration and by targeting individuals who were overweight, obese, and diagnosed with T2D. The results of this review demonstrate that engaging in some forms of exercise following a meal is effective in mitigating postprandial hyperglycemia and assisting with daily glycemic control. The improved glycemic response observed in studies using diverse exercise protocols suggests that an exercise strategy aimed at reducing blood glucose response can be readily applied without being constrained by the availability of exercise equipment or the functional capacity of an individual, as long as the exercise duration is sufficient (i.e., >30 min). Although the reduction in glucose AUC following PPE seems less affected by exercise timing, for individuals with T2D or insulin resistance, initiating exercise after the first hour post-meal may be more efficacious in mitigating 24 h mean glucose response than exercising sooner. PPE led to a more pronounced reduction in 24 h mean glucose concentrations in patients with T2D than those without the condition, suggesting its potential as a potent glucose-lowering strategy for this population.

## Figures and Tables

**Table 1:** Experimental characteristics of the included studies (*n* = 31) involving overweight (OW) individuals and individuals with obesity (OB) and type II diabetes (T2DM).

Studies	Participant Characteristics				PPE Protocols			Test Meals		Outcome Variables	Major Findings
Authors (Year)	N (Sex)	Age (Year)	BMI (kg/m^2^)	Disease Status (Fasting Glucose)	Exercise Mode and Intensity	Exercise Duration	Exercise Timing Post-Meal	Caloric Intake (Kcal)	% Energy from CHO		
Bellini et al. [47]	8	63	32	T2DM (HbA1c: 7.0%)	AE: Walking at 100 steps/min RE: A circuits of 5 exercises w/medicine ball and elastic band	AE: 30 min RE: 15 min	~30 min	310/meal	66	3 h mean glucose concentration	3 h mean glucose ↓ in AE or RE than CON
Carrillo-Arango et al. [56]	33 (24 M/9 F)	33	29	OW/OB (FG: 85 mg/dL)	8 30 s all-out cycling at 90–95% HRmax with 1 min rest period between bouts	12 min	~90 min	300/meal	100	3 h glucose AUC	3 h glucose AUC ↔ between PPE and CON
Colberg et al. [26]	12 (6 M/6 F)	61	35	T2DM (HbA1c: 7.0%)	Walking at 40% HHR	20 min	15–20 min	400–450/meal	Mixed	4 h glucose AUC	4 h glucose AUC ↔ between PPE and CON
Derave et al. [57]	7 (7 M)	45	34	OB (FG: 90 mg/dL)	Cycling at 60% VO_2_max	45 min	~60 min	516/meal	82	3 h glucose iAUC	3 h glucose iAUC ↔ between PPE and CON
DiPietro et al. [58]	10	69	30	OB (FG: 105–125 mg/dL)	Walking at 3 METs for 15 min after each meal	45 min	~30 min	32/kg/day	53	3 h mean glucose concentration; 24 h glucose AUC	3 h mean glucose ↓ in PPE than CON; 24 h glucose AUC ↓ in PPE than CON
Erickson et al. [37]	10 (2 M/8 F)	57	34	T2DM (HbA1c: 6.3%)	5 10 min cycling at 60% VO_2_max with 3 min rest between bouts	50 min	<30 min	600–1000/meal	65	2 h glucose AUC; 24 h mean glucose concentration	2 h glucose AUC ↓ in PPE than CON; 24 h mean glucose ↔ between PPE and CON
Gillen et al. [29]	7	62	31	T2DM (HbA1c: 6.9%)	10 1 min cycling at 85% HRmax with 1 min rest between bouts	10 min	~90 min	1704/day	52	2 h mean glucose concentration; 24 h mean glucose concentration	2 h mean glucose ↓ in PPE than CON; 24 h mean glucose ↔ between PPE and CON
Haxhi et al. [48]	9 (9 M)	58	30	T2DM (HbA1c: 7.0%)	Walking at 50% of HRR	40 min	~40 min	673/meal	55–60	3 h glucose iAUC; 24 h mean glucose concentration	3 h iAUC ↔ between PPE and CON; 24 h mean glucose ↔ between PPE and CON
Heden et al. [28]	13 (5 M/8 F)	49	37	T2DM (HbA1c: 7.2%)	3 sets of 10 reps in each of 8 exercises at 10 RM with ~2 min rest between sets	47 min	~45 min	832/meal	50	4 h glucose iAUC	4 h glucose iAUC ↓ in PPE than CON
Honda et al. [30]	16 (13 M/3 F)	65	24	T2DM (HbA1c: 6.9%)	Stair climbing at ~80% HRmax for 3 min after breakfast and lunch	3 min	60–120 min	460/meal	49	3 h glucose AUC	3 h glucose AUC ↓ in PPE than CON
Honda et al. [31]	7 (7 M)	70	24	T2DM (HbA1c: 7%)	Stair climbing at ~80% HRmax for 3 min after breakfast and lunch	3 min	60–120 min	522/meal	66	24 h mean glucose concentration	24 h mean glucose ↓ in PPE than CON
Kong et al. [59]	15 (15 M)	21	34	OW (NA)	4 30 s all-out cycling against 5–7.5% of BM with 4 min recovery between bouts	14 min	~90 min	NA	55	2.5 h glucose AUC	2.5 h glucose AUC ↓ in PPE than CON
Larsen et al. [49]	8 (8 M)	56	29	T2DM (HbA1c: 6.0%)	4 7 min cycling (3 min at 57% and 4 min at 98% VO_2_max with 6 min rest between bouts	46 min	<45 min	648/meal	59	4 h glucose iAUC	4 h glucose iAUC ↓ in PPE than CON
Larsen et al. [50]	9 (9 M)	60	29	T2DM (HbA1c: 7.1%)	Cycling at 50% VO_2_max	45 min	~45 min	596/meal	56	4 h glucose iAUC	4 h glucose iAUC ↓ in PPE than CON
Li et al. [38]	29 (22 M/7 F)	51	25	T2DM (HbA1c: 7.3%)	Walking at 40% HRR	20 min	<30 min	1754/day	55	2 h glucose AUC; 12 h mean glucose concentration	2 h glucose AUC ↓ in PPE than CON; 12 h mean glucose ↔ between PPE and CON
Little et al. [60]	10 (2 M/8 F)	41	36	OW/OB (FG: 5.6 mM)	HIIE: 10 1 min cycling at 90% HRmax with 1 min rest between bouts CMIE: Cycling at 65% HRmax	HIIE: 10 min CMIE: 30 min	~120 min	595/meal	70	2 h glucose iAUC; 24 h mean glucose concentration	2 h glucose iAUC ↓ in HIIE or CMIE than CON; 24 h mean glucose ↔ across HIIE, CMIE, and CON
Manders et al. [51]	9 (9 M)	57	29	T2DM (HbA1c: 7.3%)	LI: Cycling at 35% Wmax HI: Cycling at 70% Wmax	LI: 60 min HI: 30 min	<60 min	2503/day	58	4 h mean glucose; 24 h mean glucose concentration	4 h mean glucose ↔ across LI, HI, and CON; 24 h mean glucose ↓ in LI than CON
Manders et al. [32]	15 (7 M/8 F)	60	30	T2DM (HbA1c: 7.0%)	HIIE: 5 min walking at 25% HRR, 5 sets of 3 min walking at 70% HRR with 3 min recovery at 30% HRR between sets, and 5 min cooldown at 25% HRR CMIE: 5 min walking at 25% HRR, 30 min at 50% HRR, and 5 min cooldown at 25% HRR	HIIE: 40 min CMIE: 40 min	60 min	200/meal	61	50 min mean glucose concentration	50 min mean glucose ↓ in HIIE or CMIE than CON
Metcalfe et al. [52]	11 (11 M)	52	30	T2DM (HbA1c: 7.0%)	ASIE: 10 min unloaded cycling interspersed with 2 20 s all-out cycling against 5% BM; HIIE: 10 1 min cycling at 90%HRmax with 1 min recovery; CMIE: Cycling at 50% Wmax for 30 min	ASIE: 10 min HIIE: 25 min CMIE: 30 min	~30 min	2441/day	51	3 h mean glucose concentration; 24 h mean glucose concentration	3 h glucose AUC ↔ across ASIE, HIIE, CMIE, and CON; 24 h mean glucose ↓ in ASIE than CON
Moreira et al. [33]	9 (9 M)	47	29	T2DM (HbA1c: >7%)	RE-LI: 3 sets of 30 reps in each of 6 exercises at 23% 1 RM with 2 min rest between sets RE-MI: 3 sets of 16 reps in each of 6 exercises at 43% 1RM with 2 min rest between sets	RE-LI: 25 min RE-HI: 25 min	~120 min	285/meal	63	145 min glucose AUC	145 min glucose iAUC ↓ in RE-LI or RE-MI than CON
Nygaard et al. [61]	12 (8 M/4 F)	65	25	HG (HbA1c: 6.1%)	Walking at 8% and speed corresponding to RPE of 12	60 min	~30 min	2117/day	44	1 h glucose AUC; 22 h mean glucose concentration	1 h glucose AUC ↓ in PPE than CON; 22 h mean glucose ↔ between PPE and CON
Parker et al. [62]	27 (10 M/17 F)	30	30	OW/OB (FG: 4.5–5.0 mM)	HIIE: 5 min cycling at 50% Wmax, 8 1 min cycling at 100% Wmax with 1 min recovery at 50 W between bouts, and 3 min cooldown at 50% Wmax CMIE: Cycling at 50% Wmax	HIIE: 24 min CMIE: 38 min	≥60 min	500/meal	55	2 h glucose AUC; 24 h mean glucose concentration	2 h AUC ↓ in HIIE or CMIE than CON; 22 h mean glucose ↓ in HIIE or CMIE than CON
Rasmussen et al. [53]	12 (8 M/4 F)	56	29	T2DM (HbA1c: 8%)	Cycling at 40% VO_2_max	30 min	30 min	436/meal	48	4 h glucose AUC	4 h glucose AUC ↔ between PPE and CON
Sargeant et al. [63]	23 (13 M/10 F)	67	30	HG (HbA1c: 5.9%)	HIIE: 3 min walking at 3.5 mph, 10 1 min brisk and inclined walking at 90% VO_2_max with 1 min recovery at 3.5 mph between bouts, 2 min cooldown at 3.5 mph CMIE: Brisk and inclined walking at 65% VO_2_max	HIIE: 25 min CMIE: 35 min	~90 min	622–668/meal	62	6 h glucose AUC	6 h glucose iAUC ↔ across PPE-HIIE, CMIE and CON in WE and SA subgroups
Shambrook et al. [64]	10 (8 M/2 F)	50	29	OW (FG: 4.8 mM)	Ex-30 × 1: Walking at 55–70% HHR for 30 min after breakfast Ex-10 × 3: Walking at 55–70% HHR for 10 min after each meal	Ex-30 × 1: 30 min Ex-10 × 3: 30 min	30 min	NA	55	2 h glucose AUC; 24 h glucose AUC	2 h and 24 h glucose AUC ↔ between Ex-30 × 1, Ex-10 × 3, and CON
Terada et al. [54]	10 (8 M/2 F)	60	31	T2DM (HbA1c: 7.1%)	HIIE: 15 1 min inclined walking at 100% VO_2_max with 3 min recovery at 40% VO_2_max between bouts CMIE: Inclined walking at 55% VO_2_max	HIIE: 60 min CMIE: 60 min	≥60 min	600/meal	50	2 h glucose iAUC; 24 h mean glucose concentration	2 h iAUC ↔ between HIIE or CMIE and CON; 24 h mean glucose ↔ between HIIE or CMIE and CON
Van Dijk et al. [34]	60 (60 M)	60	30	T2DM (HbA1c: 7.3%)	Cycling at 35–50% Wmax	45–60 min	90–150 min	2390/day	56	24 h mean glucose concentration	24 h mean glucose ↓ in PPE than CON
Van Dijk et al. [35]	45 (45 M)	61	30	IGT and T2DM (HbA1c: 6.1–7.6%)	AE: Cycling at 50% Wmax; RE: Warm-up set at 20% BM, 3 sets of 10 reps at 40% BM in each of 2 upper-body exercises and 5 sets of 10 reps at 70% 1 RM in each of 2 lower-body exercises	AE: 45 min RE: 45 min	~150 min	2486/day	57	6 h mean glucose; 24 h mean glucose concentration	6 h mean glucose ↓ in AE or RE than CON in all subgroups; 24 h mean glucose ↓ in AE or RE than CON in IGT, OGLM, and INS subgroups
van Dijk et al. [36]	30 (30 M)	60	30	T2DM (HbA1c: 7.0–7.4%)	Ex-Daily: Cycling at 50% Wmax for 30 min every 24 h over a 48 h period Ex-Nondaily: Cycling at 50% Wmax for 60 min over a 48 h period	Ex-Daily: 30 min/24 h Ex-Nondaily: 60 min/48 h	~90 min	2462/day	55	48 h mean glucose concentration	48 h mean glucose ↓ in Ex-Daily or Ex-Nondaily than CON
van Dijk et al. [55]	20 (20 M)	64	30	T2DM (HbA1c: 6.9%)	Ex-45 × 1: Cycling at 6 METs for 45 min after breakfast Ex-15 × 3: Cycling at 3 METs 15 min after each meal	Ex-45 × 1: 45 min Ex-15 × 3: 45 min	~45 min	2342/day	50	24 h mean glucose concentration	24 h mean glucose ↓ in Ex-45 × 1 than CON, but ↔ between Ex-15 × 3 and CON
Zhang et al. [39]	20 (20 M)	23	27 (OB by WHO Guidelines)	OW/OB (HbA1c: 5.3%)	Ex-20 min post: Walking at 50% VO_2_max ~20 min post-meal Ex-40 min post: Walking at 50% VO_2_max ~40 min post-meal	Ex-20 min post: 30 min Ex-20 min post: 30 min	~20 min and ~40 min	1970–1980/day	49.7–51.2	4 h glucose iAUC; 24 h mean glucose concentration	4 h iAUC ↓ in Ex-20 min post or Ex-40 min post than CON; 24 h mean glucose ↔ between Ex-20 min post or Ex-40 min post and CON

Note: Thirty-one studies were selected for the meta-analysis. Values for age, BMI, and fasting glucose are the group means extracted from the studies. PPE: Postprandial exercise; CON: Time-matched control condition; ASIE: All-out sprint interval exercise; HIIE: High-intensity interval exercise; CMIT: Continuous moderate-intensity exercise; AE: Aerobic exercise; RE: Resistance exercise; MET: Metabolic equivalent; HHR: Heart rate reserve; W: Power in watts; BM: Body mass; LI: Lower intensity; MI: Moderate intensity; HI: High intensity; AUC: Area under the curve; iAUC: Incremental AUC; WE: White Europeans; SA: South Asians; IGT: Impaired glucose tolerance; OGLM: Oral glucose-lowering medication; INS: Insulin treatment; ↓: Significantly different, *p* < 0.05; ↔: not significantly different, *p* ≥ 0.05.

**Table 2:** Results of quality assessment for the included studies (*n* = 31).

Studies	Q1	Q2	Q3	Q4	Q5	Q6	Q7	No. of “Y”
Bellini et al. [47]	Y	Y	Y	N	Y	Y	Y	6
Carrillo-Arango et al. [56]	Y	Y	Y	Y	Y	Y	Y	7
Colberg et al. [26]	Y	Y	Y	N	Y	Y	Y	6
Derave et al. [57]	Y	Y	Y	N	Y	Y	Y	6
DiPietro et al. [58]	Y	Y	Y	N	Y	Y	Y	6
Erickson et al. [37]	Y	Y	Y	N	Y	Y	Y	6
Gillen et al. [29]	Y	Y	N	N	Y	Y	Y	5
Haxhi et al. [48]	Y	Y	Y	N	Y	Y	Y	6
Heden et al. [28]	Y	Y	Y	N	Y	Y	Y	6
Honda et al. [30]	Y	Y	N	N	Y	Y	Y	5
Honda et al. [31]	Y	Y	N	Y	Y	Y	Y	6
Kong et al. [59]	Y	Y	Y	Y	Y	Y	Y	7
Larsen et al. [49]	Y	Y	Y	N	Y	Y	Y	6
Larsen et al. [50]	Y	Y	Y	N	Y	Y	Y	6
Li et al. [38]	Y	Y	Y	N	Y	Y	Y	6
Little et al. [60]	Y	Y	N	N	Y	Y	Y	5
Manders et al. [51]	Y	Y	Y	N	Y	Y	Y	6
Manders et al. [32]	Y	Y	Y	Y	Y	Y	Y	7
Metcalfe et al. [52]	Y	Y	Y	Y	Y	Y	Y	7
Moreira et al. [33]	Y	Y	N	N	Y	Y	Y	5
Nygaard et al. [61]	Y	Y	Y	Y	Y	Y	Y	7
Parker et al. [62]	Y	Y	Y	N	Y	Y	Y	6
Rasmussen et al. [53]	Y	Y	N	N	Y	Y	Y	5
Sargeant et al. [63]	Y	Y	Y	Y	Y	Y	Y	7
Shambrook et al. [64]	Y	Y	Y	N	Y	Y	Y	6
Terada et al. [54]	Y	Y	Y	N	Y	Y	Y	6
Van Dijk et al. [34]	Y	Y	Y	N	Y	Y	Y	6
Van Dijk et al. [35]	Y	Y	Y	N	Y	Y	Y	6
van Dijk et al. [36]	Y	Y	Y	N	Y	Y	Y	6
van Dijk et al. [55]	Y	Y	Y	N	Y	Y	Y	6
Zhang et al. [39]	Y	Y	Y	Y	Y	Y	Y	7
No. of “Y”	31	31	25	8	31	31	31	Overall Mean = 6.1

Note: Y: “Yes” to the question; N: “No” to the question. Q1–Q7 are the questions that assess research quality and can be found in the Methods section.

**Table 3:** Results of subgroup analysis on the glucose-lowering effect based on exercise duration, exercise timing post-meal, and the disease status of participants.

Factors	Subgroup Categories	Postprandial Glucose AUC			24 h Mean Glucose Levels		
		Hedges’ g (PPE vs. CON)	Test for Subgroup Homogeneity		Hedges’ g (PPE vs. CON)	Test for Subgroup Homogeneity	
			Chi-Square Q-Statistic	*p* Value		Chi-Square Q-Statistic	*p* Value
Exercise Duration	≤30 min	−0.209	4.361	0.037 *	−0.225	1.994	0.158
	>30 min	−0.450			−0.401		
Exercise Timing Post-Meal	<60 min	−0.331	0.044	0.833	−0.163	4.463	0.035 *
	≥60 min	−0.307			−0.430		
Disease Status of Participants	Without T2DM	−0.239	1.194	0.275	−0.126	4.104	0.043 *
	With T2DM	−0.368			−0.405		

Note: * Significantly different between the two subgroups (*p* < 0.05).

## Data Availability

Not applicable.
